# Correlation between SUA and prognosis in CHF patients after revascularization

**DOI:** 10.5937/jomb0-45322

**Published:** 2024-04-23

**Authors:** Bo Miao, Jing Wu, Wang Jiao, Li Yanxin, Yingxiao Da, Wang Dong, Bei Gao

**Affiliations:** 1 Xingtai Third Hospital, Cardiovascular Intensive Care Unit, Xingtai, China; 2 Xingtai Third Hospital, Department 2 of Cardiology, Xingtai, China; 3 Xingtai Third Hospital, Department 1 of Cardiology, Xingtai, China; 4 The Second Hospital of Hebei Medical University, Department of Cardiology, Shijiazhuang, China; 5 Xingtai Central Blood Station, Xingtai, China

**Keywords:** serum uric acid, chronic heart failure, revascularization, cardiac function, prognosis, correlation, mokraćna kiselina, hronična srčana insuficijencija, revaskularizacija, srčana funkcija, prognoza, korelacija

## Abstract

**Background:**

To explore the correlation between serum uric acid (SUA) and prognosis in patients with chronic heart failure (CHF) after revascularization.

**Methods:**

A total of 126 patients with CHF undergoing revascularization [coronary artery intervention (PCI) or coronary artery bypass grafting (CABG)] in the hospital were enrolled as CHF group between December 2021 and October 2022, while 126 healthy controls during the same period were enrolled as healthy control group. The levels of SUA, inflammatory factors and cardiac function in the two groups were detected. The correlation between SUA level and inflammatory factors, cardiac function levels was analyzed. All patients in CHF group were followed up for 6 months to observe prognosis. The differences in the above indexes among patients with different prognosis were compared. The risk factors of prognosis were analyzed by multivariate Logistic regression analysis, and their predictive value for prognosis was evaluated by ROC curves analysis.

## Abstract (sr)

**Uvod:**

Cilj je bio da se istraži korelacija između serumske mokraćne kiseline (SUA) i prognoze kod pacijenata sa hronićnom srčanom insuficijencijom (CHF) nakon revaskularizacije.

**Metode:**

Ukupno 126 pacijenata sa CHF koji su bili podvrgnuti revaskularizaciji [koronarne arterijske intervencije (PCI) ili koronarne arterijske premosnice (CABG)] u bolnici je upisano kao grupa za CHF između decembra 2021. i oktobra 2022. godine, dok je 126 zdravih kontrola tokom istog perioda su upisani kao zdrava kontrolna grupa. Detektovani su nivoi SUA, inflamatornih faktora i srčane funkcije u dve grupe. Analizirana je korelacija između nivoa SUA i inflamatornih faktora i nivoa srčane funkcije. Svi pacijenti u grupi sa CHF su praćeni 6 meseci da bi se posmatrala prognoza. Upoređene su razlike u navedenim indeksima kod pacijenata sa različitom prognozom. Faktori rizika prognoze analizirani su multivarijantnom logističkom regresionom analizom, a njihova prediktivna vrednost za prognozu procenjena je analizom ROC krive.

**Rezultati:**

SUA, IL-6, TNF-a, NT-proBNP i LVEDD u grupi sa CHF su bili viši od onih u zdravoj kontrolnoj grupi, dok je LVEF bio niži u odnosu na zdravu kontrolnu grupu (P<0,05). Među 126 pacijenata u roku od 6 meseci nakon operacije, bilo je 24 slučaja (19,05%) sa neželjenim srčanim događajima (loša prognoza). SUA, IL-6, TNF-a, NT-proBNP i LVEDD kod pacijenata sa lošom prognozom bili su veći od onih sa dobrom prognozom, dok je LVEF bio niži od onih sa dobrom prognozom (P<0,05). Nivo SUA je bio u pozitivnoj korelaciji sa IL-6, TNF-a, NT-proBNP i LVEDD (r=0,283, 0,292, 0,322, 0,355, P<0,05), dok je u negativnoj korelaciji sa LVEF (r=-0,368, P<0,05). Visok nivo SUA i nizak LVEF su bili nezavisni faktori rizika prognoze (OR=1,486, 0,678, P<0,05). I SUA i LVEF su bile prognostičke vrednosti (AUC=0,805, 0,809, P<0,05).

**Zaključak:**

Nivo SUA je značajno povećan kod pacijenata sa CHF, što je nezavisan faktor rizika za lošu prognozu kod pacijenata sa CHF nakon revaskularizacije, sa visokom prognostičkom vrednošću.

## Introduction

Chronic heart failure (CHF) is a complex clinical syndrome, which can be caused by hypertension,coronary heart disease, etc. Coronary heart disease is the most common disease, which develops slowly. It is the end stage manifestation of cardiovascular disease, and is related to multiple factors such as decreased endothelial cell function, inflammatory reaction and oxidative stress. It is also one of the causes of death of such patients, with a high Case fatality rate [1] [2]. Revascularization is an effective method for treating CHF, which improves cardiac hemodynamics, improves cardiac load, alleviates clinical symptoms, and controls malignant progression of the disease. However, there is still a high incidence of adverse prognosis after surgery. Early prediction of prognosis can effectively guide clinical early intervention, improve patient prognosis, and improve patient quality of life [3] [4]. The prognosis of patients with heart failure (CHF) after revascularization depends on many factors. The markers of poor prognosis included advanced age, underlying diseases, cardiac insufficiency and complications, while the good prognosis was characterized by good preoperative cardiac function, smooth rehabilitation process and no serious complications. Physicians consider a combination of these factors, but because each patient's situation is unique, prognosis assessment needs to be individualized and closely monitored. In the past, many studies have said that [5] [6] changes in serum uric acid (SUA) levels are independently related to the incidence rate and mortality of cardiovascular diseases, and some studies have also shown that [7] Hyperuricemia (HUA) is associated with poor prognosis of elderly CHF, and the risk of rehospitalization of patients with HUA is significantly increased. Moreover, SUA can also cause inflammatory and oxidative stress reactions. Therefore, the author considers whether detecting SUA levels after CHF revascularization surgery can predict patient prognosis. However, there is still controversy in domestic and international research. Based on this, this study explores the correlation between serum SUA levels and the prognosis of CHF patients undergoing revascularization surgery, providing evidence-based evidence for clinical practice.

## Materials and methods

### General information

126 CHF patients who underwent revascularization surgery in our hospital from December 2021 to October 2022 were selected as the CHF group, and 126 healthy individuals in the same period were selected as the healthy control group. Inclusion criteria: ① All CHF groups met the diagnostic criteria for CHF [8]; The physical examination results of the healthy control group were all normal; Those in the CHF group who meet the surgical indications and have no difference in at least two SUA tests before this surgery; ② The patient and their family members have informed and signed a written consent form regarding the content of this study. Exclusion criteria: ① Patients with concomitant malignant tumors; ② Those with acute and chronic infectious diseases; ③ Patients with concomitant hematological diseases; Patients with pulmonary or infectious heart disease; Patients with other types of heart diseases; Individuals with autoimmune system disorders; Individuals with gout and taking medication that may affect experimental results; Individuals with ahistory of major surgeries within the past year; The loser of this surgery; Missing visitors. CHF group, male/female=75/51 cases, age 54–72 years, average (62.89 ± 4.09) years old, body mass index (BMI) (21.98 ± 2.16) kg/m^2^; The New York Heart Association classification: II/III/IV=40/52/34 cases. A healthy control group, male/female=68/58 cases, aged 56–72 years, with an average age of (63.68 ± 3.42) years and a BMI of (22.12 ± 2.37) kg/m^2^. There was no difference in general information (age, gender, BMI) between the two groups (*P*>0.05).

## Method

### Treatment method

Choose coronary intervention (PCI) or Coronary artery bypass surgery (CABG) according to the patient's condition. After surgery, they were given aspirin, statins and other drugs for a long time, and asked patients to control the original basic diseases, such as diabetes, hypertension, etc.

### Serological indicator testing

Relevant serum tests were performed in the CHF group at 1 d after surgery, and serum specimens were kept for investigation in the healthy control group during physical examination. All fasting venous blood was drawn, serum was separated (3500 r/min, 10 min, radius 8 cm) and stored at -80°C for investigation of Inter leukin-6 (IL-6), Tumor necrosis factor-α (TNF-α), N-terminal pro-brain natriuretic peptide (NT-proBNP)).

### Cardiac function test

One day after operation, color Doppler ultrasound was used to detect cardiac function, and left ventricular end diastolic diameter (LVEDD) and left ventricular Ejection fraction (LVEF) were recorded.

### Follow up prognostic evaluation

Patients in the CHF group were followed up for 6 months to observe the occurrence of adverse cardiac events, including exacerbation of heart failure, re hospitalization, malignant arrhythmia, cardiac death, or all cause death.

### Observation indicators

(1) Comparison of serological indicators and cardiac function index between the two groups; (2) Recording of the prognosis of the CHF group and comparison of serological indicators and cardiac function index of CHF patients with different prognosis; (3) Pearson method analysis of correlation between SUA and inflammatory factors and cardiac function index; (4) Multi-factor logistic regression analysis of risk factors affecting the prognosis of CHF and ROC curve analysis for factor predictive value assessment.

### Statistical analysis

SPSS 22.0 statistical software was used for data analysis. The measurement data met the normal distribution and were expressed as (x̄±s) with t-test, while the count data were expressed as rates with x^2^ test. Factor predictive value assessment was performed, and P<0.05 suggested statistical significance.

## Results

### Comparison of serological and cardiac function indicators between the two groups

**Figure 1:**
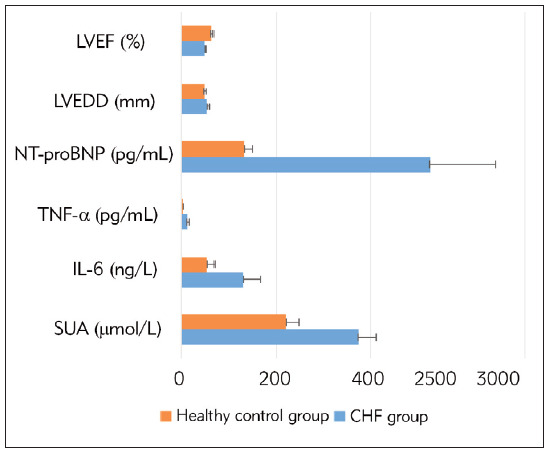
Comparison of serological indicators between the two groups.

Serum SUA, IL-6, TNF-α, NT-proBNP levels and cardiac function index LVEDD were higher in the CHF group than in the healthy control group, and cardiac function index LVEF was lower than in the healthy control group (P<0.05). See Table 1, Figure 1.

**Table 1:** Comparison of serological indicators between the two groups (x̄±s).

Group	Cases	SUA (μmol/L)	IL-6 (ng/L)	TNF-α<br>(pmol/L)	NT-proBNP<br>(pg/mL)	LVEDD (mm)	LVEF (%)
CHF group	126	374.24±37.89	129.97±35.17	13.01±4.52	2526.14±387.63	55.29±5.11	50.12±3.59
Healthy control<br>group	126	221.62±26.54	54.87±15.26	4.18±1.29	132.58±15.74	48.59±4.23	63.78±3.26
t		37.033	21.989	21.086	69.256	11.337	31.620
P		0.000	0.000	0.000	0.000	0.000	0.000

### Comparison of serological and cardiac function indicators in CHF patients with different prognosis

**Figure 2:**
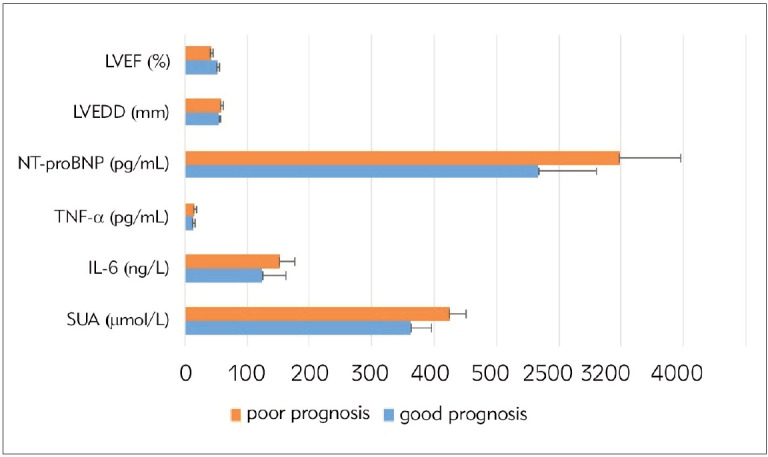
Comparison of serological and cardiac function indicators in CHF patients with different prognoses.

Adverse cardiac events occurred in 24 patients within 6 months after surgery in 126 patients, with an incidence of 19.05%, and were judged to have a bad prognosis. Serum SUA, IL-6, TNF-α, NT-proBNP levels and cardiac function index LVEDD were higher in those with bad prognosis than in those with favorable prognosis, and cardiac function index LVEF was lower than in those with favorable prognosis (P<0.05). See Table 2, Figure 2.

**Table 2:** Comparison of serological and cardiac function indicators in CHF patients with different prognoses (x̄±s).

Group	Cases	SUA (μmol/L)	IL-6 (ng/L)	TNF-α (pmol/L)	NT-proBNP<br>(pg/mL)	LVEDD (mm)	LVEF (%)
Good prognosis	102	362.25±33.69	124.65±38.47	12.58±3.93	2367.89±742.16	54.68±2.98	52.14±4.36
Poorprognosis	24	425.21±25.96	152.58±24.78	14.77±4.36	3198.71±725.69	57.87±2.79	41.53±3.52
t		16.616	6.851	4.188	8.985	8.772	21.254
P		0.000	0.000	0.000	0.000	0.000	0.000

### Correlation analysis

SUA levels in CHF patients were positively correlated with IL-6, TNF-α, NT-proBNP levels and LVEDD index (*r*=0.283, 0.292, 0.322, 0.355, P<0.05) and negatively correlated with LVEF index (r=-0.368, P<0.05).

### Multi-factor logistic regression analysis of risk factors affecting prognosis of CHF

High serum SUA levels and low LVEF were independent risk factors affecting the prognosis of CHF (OR=1.486, 0.678, P<0.05). See Table 3.

**Table 3:** Multi-factor Logistic Regression Analysis of Risk Factors Affecting Prognosis of CHF.

Indicators		SE	Wald 2	OR	95%CI	P
SUA	0.396	0.158	6.282	1.486	1.090~2.025	0.013
IL-6	0.289	0.169	2.924	1.335	0.859~1.859	0.088
TNF-α	0.315	0.179	3.097	1.370	0.965~1.946	0.080
NT-proBNP	0.339	0.185	3.358	1.404	0.977~2.017	0.068
LVEDD	0.369	0.195	3.581	1.446	0.987~2.120	0.059
LVEF	-0.389	0.145	7.197	0.678	0.510~0.901	0.008

### ROC curve analysis

**Figure 3:**
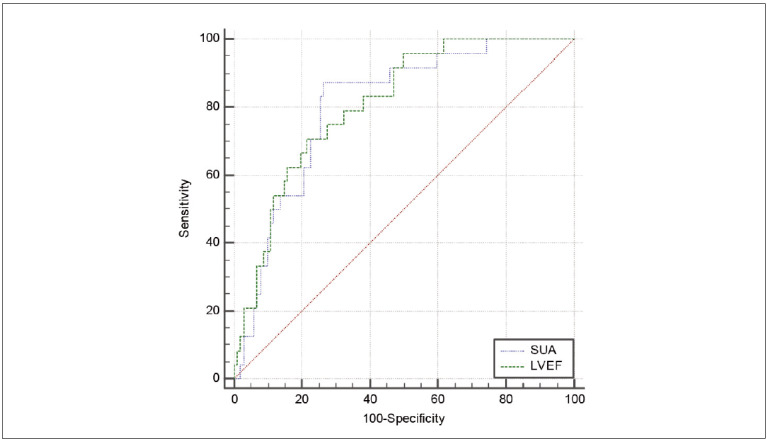
ROC curve analysis graph.

SUA level and LVEF index were all of value in predicting prognosis after CHF hemodynamic reconstruction (AUC=0.805, 0.809, P<0.05). See Figure 3, Table 4.

**Table 4:** ROC curve analysis results.

Indicators	AUC	95%CI	Truncation value	Sensitivity (%)	Specificity (%)	P
SUA	0.805	0.725~0.870	>405.07 μmol/L	87.50	73.53	0.000
LVEF	0.809	0.730~0.874	42.20%	70.83	78.43	0.000

## Discussion

CHF refers to the serious terminal stage of various cardiovascular diseases. It is a series of ischemia and hypoxia symptoms caused by various reasons, such as abnormal cardiac systolic or diastolic function, increased cardiac pre and post load, failure of cardiac pumping function, decreased cardiac output, insufficient basic blood flow of body tissues and organs, and high mortality and disability rate [9]. Revascularization is an effective method to improve patient symptoms, but its incidence of poor prognosis is still high [10]. Therefore, early prediction and prevention are necessary to improve patient prognosis. HUA is closely related to poor prognosis of cardiovascular disease, so this study explored the correlation between serum SUA level and prognosis after revascularization of CHF.

The results of this study showed that serum SUA, IL-6 and TNF-α levels were higher in the CHF group than in the healthy control group, suggesting a state of high SUA levels and high inflammatory factor levels in CHF patients, and the study showed that SUA, IL-6 and TNF-α levels were higher in patients with poor prognosis CHF than in patients with good prognosis CHF, similar in part to previous studies [11], suggesting the involvement of SUA and inflammatory responses in the development of CHF disease may be related to their prognosis after CHF revascularization. The reason for this may be that xanthine oxidase (XO) is one of the key enzymes in the metabolism of SUA, and an increased level of SUA results in excessive activation of XO, which induces the production of reactive oxygen species and the release of oxygen radicals, generating oxidative stress and damaging vascular endothelial cells, while promoting the secretion of inflammatory factors, such as IL-6 and TNF-α, causing excessive inflammation in the body. The vicious cycle of excessive reaction, damage to vascular endothelial cells and exacerbation of oxidative stress leads to progressive hypertrophy, hyperplasia, fibrosis and other abnormal manifestations of cardiomyocytes, prompting ventricular remodeling and causing a decrease in myocardial contractility [12] [13]. Correlation studies have also shown that SUA is associated with IL-6, TNF-α There is a positive correlation between levels. Moreover, endothelial cell damage can also promote platelet aggregation, promote arteriosclerosis, and accelerate the occurrence and malignant development of CHF [14]. NT-proBNP is an inactive N-terminal fragment of brain natriureticpeptide Prohormone after splitting, which is mainly secreted when the ventricular cell load increases before and after splitting, and its level change can better predict the occurrence of CHF and the assessment of its severity [15]. LVEDD and LVEF index are ultrasound indicators commonly used in clinic to assess cardiac systolic function, and their value changes are related to the condition of CHF [16]. In this study, the NT-proBNP level and LVEDD index in the CHF group were higher than those in healthy controls, and the LVEF index was lower than those in healthy controls, and the NT-proBNP level and LVEDD index in patients with poor prognosis were higher than those with good prognosis, and the LVEF index was lower than those with good prognosis, suggesting that poor prognosis is associated with impaired cardiac function, with abnormal cardiac function being a key contributing factor to unfavorable outcomes. The correlation study showed that SUA was positively correlated with NT-proBNP level and LVEDD index, and negatively correlated with LVEF index, suggesting that changes in SUA were associated with changes in cardiac function, and multi-factor Logistic regression analysis showed that high serum SUA level and low LVEF index were independent risk factors for poor prognosis [17]. Previous studies have also mentioned SUA as a significant predictor of all causes, cardiovascular mortality and heart failure hospitalization, and ROC curve analysis in this study also showed that SUA has value in predicting poor prognosis after reconstructive hemodynamic surgery in CHF. In the study by Doehner et al. [18], it was also shown that as SUA levels increased, CHF survival decreased and the rate of composite endpoint events (rehospitalisation for exacerbation of heart failure, malignant arrhythmia, cardiac death or all-cause death) increased. All suggest that SUA is closely associated with prognosis after CHF revascularisation.

In summary, serum SUA levels are significantly increased in CHF patients and are an independent risk factor for poor prognosis after revascularization in CHF patients, with high predictive value for prognosis.

## Dodatak

### Conflict of interest statement

All the authors declare that they have no conflict of interest in this work.
